# Extending susceptible-infectious-recovered-susceptible epidemics to allow for gradual waning of immunity

**DOI:** 10.1098/rsif.2023.0042

**Published:** 2023-09-13

**Authors:** Mohamed El Khalifi, Tom Britton

**Affiliations:** Department of Mathematics, Stockholm University, Stockholm, Sweden

**Keywords:** SIRS epidemic, immunity waning, vaccination, herd immunity

## Abstract

Susceptible-infectious-recovered-susceptible (SIRS) epidemic models assume that individual immunity wanes in one leap, from complete immunity to complete susceptibility. For many diseases immunity on the contrary wanes gradually, something that has become even more evident during COVID-19 pandemic where also recently infected have a reinfection risk, and booster vaccines are given to increase immunity. Here, a novel mathematical model is presented allowing for the gradual decay of immunity following linear or exponential waning functions. The two new models and the SIRS model are compared assuming all three models have the same cumulative immunity. When no intervention is put in place, we find that the long-term prevalence is higher for the models with gradual waning. If aiming for herd immunity by continuous vaccination, it is shown that larger vaccine quantities are required when immunity wanes gradually compared with results obtained from the SIRS model, and this difference is the biggest for the most realistic assumption of exponentially waning of immunity. For parameter choices fitting to COVID-19, the critical amount of vaccine supply is about 50% higher if immunity wanes linearly, and more than 150% higher when immunity wanes exponentially, when compared with the classic SIRS epidemic model.

## 1. Introduction

When considering infectious disease outbreaks over a long time horizon, waning of immunity, from disease exposure or vaccination, is known to play an important role. This has been considered in epidemic models for many years, and the most well-studied model is the susceptible-infectious-recovered-susceptible (SIRS) epidemic model, where all individuals are classified as being either susceptible, infectious or recovered (implicitly assuming also being immune), and where individuals eventually lose their immunity and go back to being susceptible after some time, e.g. [1]. The simplest form of this epidemic model, defined by differential equations, assumes that recovered individuals go back to being susceptible at a constant rate, thus implying that immunity at the *community level* wanes continuously. However, the SIRS model does *not* allow for partially immune individuals or that immunity wanes gradually at the *individual level*: each individual is either completely immune or fully susceptible.

Vaccines are designed to stimulate an immune response that is similar to the response elicited by natural infection, while avoiding the actual disease. However, vaccines are not perfect in general and do not confer lifelong protection. The term *leaky vaccine* is commonly used to describe a vaccine that fails in degree of protection, and *all-or-nothing vaccine* for a vaccine that fails in take [2]. The present work is motivated by vaccines that exhibit a failure in duration, commonly referred to as *waning vaccines*, which for example can be modelled according to the standard SIRS model [3].

During COVID-19 pandemic, but also prior to this, it has become evident that *individual immunity* (to infection) is not a binary property, but rather that individual immunity wanes gradually over time and can later be boosted either by vaccination or natural infection (see e.g. [4,5] for empirical evidence, where measured antibody levels data were used to find that individual immunity decays gradually over time, see also the systematic review [6]). Recently, [7] addressed this gradual waning of immunity as well as the importance of early initiation of boosting campaigns in controlling the epidemic. This waning of the protection over time has been shown to correlate with the neutralizing antibodies produced since the primary vaccine doses, and hence the importance of booster doses [8]. Although the antibody titres decay over time, it is not *known* if immunity wanes gradually and if so, in what form. On the other hand, SIRS epidemic models can never have a group of individuals having lost about half of their immunity, but the models do allow 50% of the community to be completely immune and 50% be completely susceptible. However, these situations are quite different, in particular when additional individuals get infected.

This continuous waning of immunity has received less attention in the literature compared with SIRS models. Models that do consider gradual immunity waning often comprise both ordinary differential equations (ODEs) and partial differential equations (PDEs), e.g. [9–11], or [8] using micro-simulation techniques with individual immunity waning. We refer to [12,13] and the references therein for an overview of such models and their mathematical analysis.

In the current paper, we define and analyse an epidemic model similar in spirit for the gradual waning of immunity. Here individuals sequentially lose a portion of their immunity in each step, up to a total of *k* steps when all immunity is lost. For large *k* this approximates the situation where immunity drops continuously in time, and we consider both the situation where immunity drops linearly and when immunity drops exponentially (the latter seemingly more biologically reasonable). We call our model the SIR^(*k*)^S epidemic model since there now are *k* immunity (recovered) levels, *k* = 1 being the classic SIRS model. It is worth pointing out that the current paper considers immunity to infection, and not immunity to severe disease and how this wanes. The latter is also an important area which has received attention in several other papers (cf. [14–16]). In addition, our model is oversimplified and neglects several aspects of immunity and the focus lies only on its gradual decline. Similar ODE models have been introduced [10,11] allowing to distinguish not only between immunity states but also between infected states according to which immunity class they come from. Yet, our analyses and results go beyond what has been done in these papers.

The three models, the classical SIRS model with a sudden complete drop of immunity, linear decay of immunity and exponential immunity decay, are calibrated by assuming the same cumulative amount of immunity. So for instance, the SIRS model with, on average, 1 year complete immunity, and then returning to complete susceptibility, is compared with the linear immunity decay model taking two years from complete immunity to full susceptibility. For each model, we derive expressions for the basic reproduction number *R*_0_ and the steady-state prevalence (endemic level) if no preventive measures are put in place, helping to understand the equilibrium of endemic infections with immunity waning [17]. We also derive the critical amount of vaccine supply needed to reach and maintain herd immunity, for each of the three models. Our models do not allow for differences between infection-induced immunity and vaccine-induced immunity, but this could be considered by introducing more compartments to distinguish between vaccinated and recovered populations.

To the best of our knowledge, none has yet put attention on how the implied long-term prevalence compares with the standard SIRS one and also how the amount of vaccines needed for a stable herd immunity varies when the disease can invade both partially and fully susceptible individuals.

Our main conclusion shows that the situation is worse for the more realistic models allowing for gradual waning of immunity compared with the classic SIRS model: even though the three models share the same *R*_0_ the models with gradual waning will result in higher prevalence (endemic level) if no preventive measures are put in place, and more vaccine supply (or other preventive measures) are needed to reach a steady herd immunity, implying that vaccination policies (or other preventive measures) based on the SIRS epidemic model may lead to an incorrect sense of security. Among the two studied models for immunity waning, linear and exponential decay, the more realistic exponential decay shows the biggest difference (of endemic prevalence and critical amount of vaccine supply) compared with the classic SIRS model. As a direct consequence, if in a real situation immunity wanes gradually (e.g. exponentially), and the SIRS model is used for determining the required amount of vaccines, then our results show that the SIRS model *underestimates* the critical amount of vaccines needed. It is easy to show that the opposite holds in the situation where the truth is that immunity wanes in one jump, whereas a model with gradual waning is used in the analysis. The latter situation seems more unlikely, but could be of relevance, e.g. when a new strain enters, with the effect that immunity makes a more sudden drop.

## 2. Model and main results

### 2.1. Formulation of the models

All three models assume that (i) immunity from vaccination as well as disease exposure initially confer complete immunity and (ii) that immunity from vaccination wanes in the same way as immunity from disease exposure. Further, infectious individuals have infectious contacts at rate *β* and recover (and become fully immune) at rate *γ*. The differences between the models lie in how immunity wanes, and what is the rate of getting infected for a partially immune in relation to a fully susceptible.

**Figure 1.**
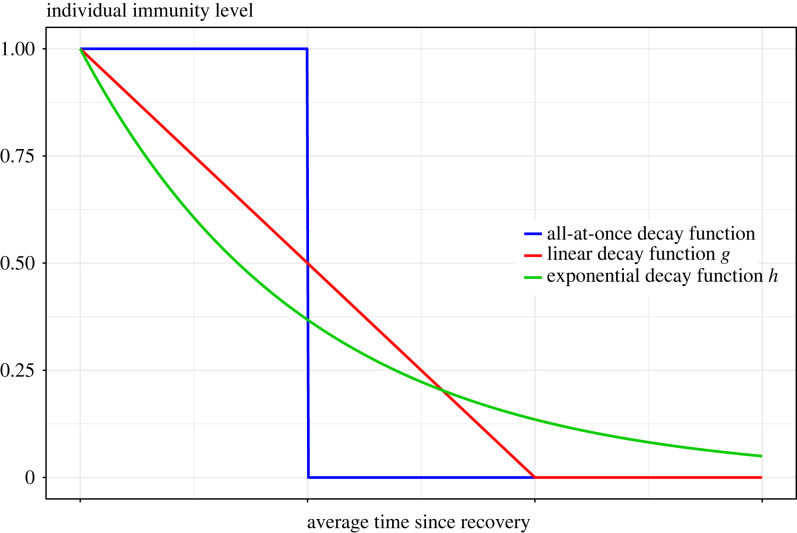
Different functions of waning of immunity on individual level: (blue) all-at-once decay (taking place at its expected time-point), (red) linear decay function *g*(*u*) = 1 − *ω*/2 *u* if 0 ≤ *u* ≤ 2/*ω*, and *g*(*u*) = 0 otherwise, and (green) exponential decay function *h*(*u*) = exp (−*ωu*), *u* ≥ 0. All three models having the same average cumulative immunity.

Figure 1 illustrates the immunity waning for the classic SIRS epidemic (assuming waning happens at its expected time-point) and for the models with linear and exponential decay of waning.

#### 2.1.1. The classic SIRS epidemic

**Figure 2.**
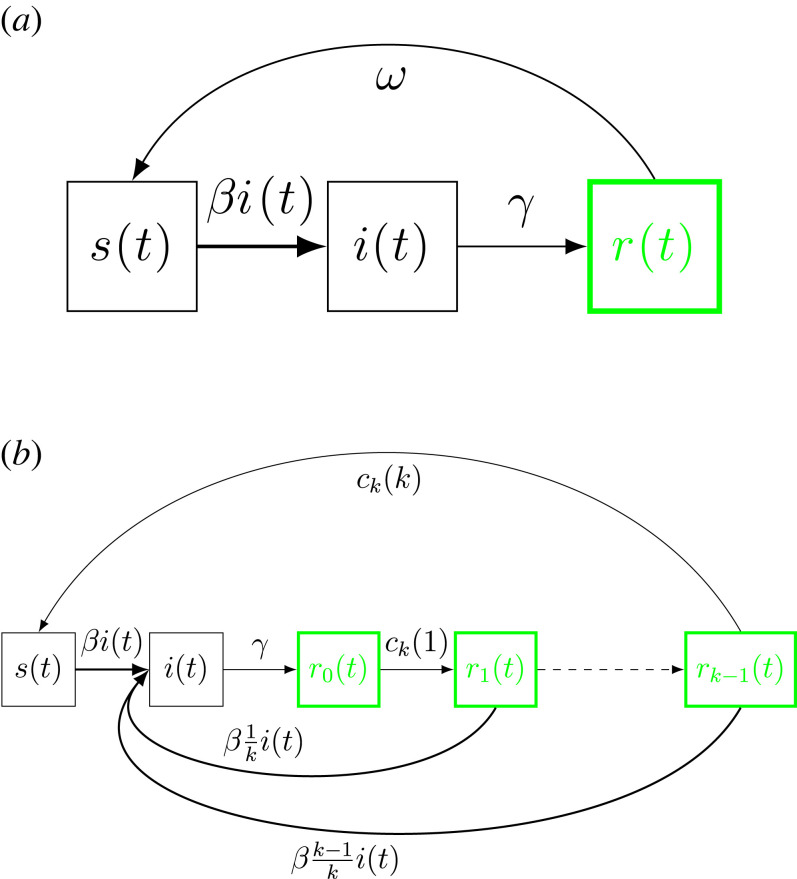
(*a*) Diagram of the standard SIRS epidemic model. (*b*) Diagram of the SIR^(*k*)^S epidemic model. The green boxes represent the different classes of partially immune states.

The classic SIRS epidemic model assumes that immunity drops from complete immunity to complete susceptibility in one single step at a constant rate *ω* (so the mean duration of full immunity equals *ω*^−1^) [1]. The model is illustrated in figure 2*a*, where *s*(*t*), *i*(*t*) and *r*(*t*) denote community fractions of susceptible, infectious and recovered (= immune) individuals at time *t*, respectively. The model is defined by the following three differential equations:

2.1 $$\begin{array}{rl} s^{\prime }(t) & =\mu -\beta s(t)i(t)+\omega r(t)-\mu s(t),\\ i^{\prime }(t) & =\beta s(t)i(t)-(\gamma +\mu )i(t)\\ \mathrm{and}\;\;r^{\prime }(t) & =\gamma i(t)-(\omega +\mu )r(t), \end{array}\}$$

where *β* is the transmission rate, *γ*^−1^ the mean contagious period and *μ*^−1^ is the life expectancy.

#### 2.1.2. The classic SIR^(*k*)^S epidemic with linear/exponential waning

Our new model, denoted the SIR^(*k*)^S epidemic model, instead assumes that immunity wanes sequentially in *k* steps (for some large *k*), as illustrated in figure 2*b* (*k* = 1 gives the classic SIRS epidemic). The linear version does so by choosing the *k* down-jumps and their corresponding rates such that the decay mimics a linear decay, and the exponential version chooses down-jumps and rates to mimic exponential decay, and both models do this in a way such that the cumulative immunity equals *ω*^−1^ (independent of *k*) just like the SIRS model. The new model is illustrated in figure 2*b* and defined in detail with *k* + 2 differential equations in §4. There *r*_0_(*t*) denotes the community fraction having no susceptibility, *r*_1_(*t*) the community fraction having gained one level of susceptibility, and so on, and *r*_*k*−1_(*t*) the fraction having susceptibility level *k* − 1 being the last step before becoming completely susceptible.

In [18], similar waning functions were used to model the vaccination efficacy over time while they estimate the cost-effective vaccination strategy against tuberculosis.

We let SIR^(∞)^S denote the model being the limit of the SIR^(*k*)^S model as *k* goes to ∞ (in our illustrations we use *k* = 1000). This limiting model converges to an ODE-PDE system with three equations, which has been introduced and studied in, e.g. [9,12,19], see §4.8.

#### 2.1.3. Introducing vaccination

As mentioned earlier, we assume that vaccine as well as infection initially give full immunity, and that the two immunities wane in the same way.

In the classic SIRS model each individual is either fully susceptible or completely immune at any given point in time, and if this immune status is known, it of course only makes sense to vaccinate among the fully susceptible individuals at some rate *η* (why waste vaccines on fully immune?).

**Figure 3.**
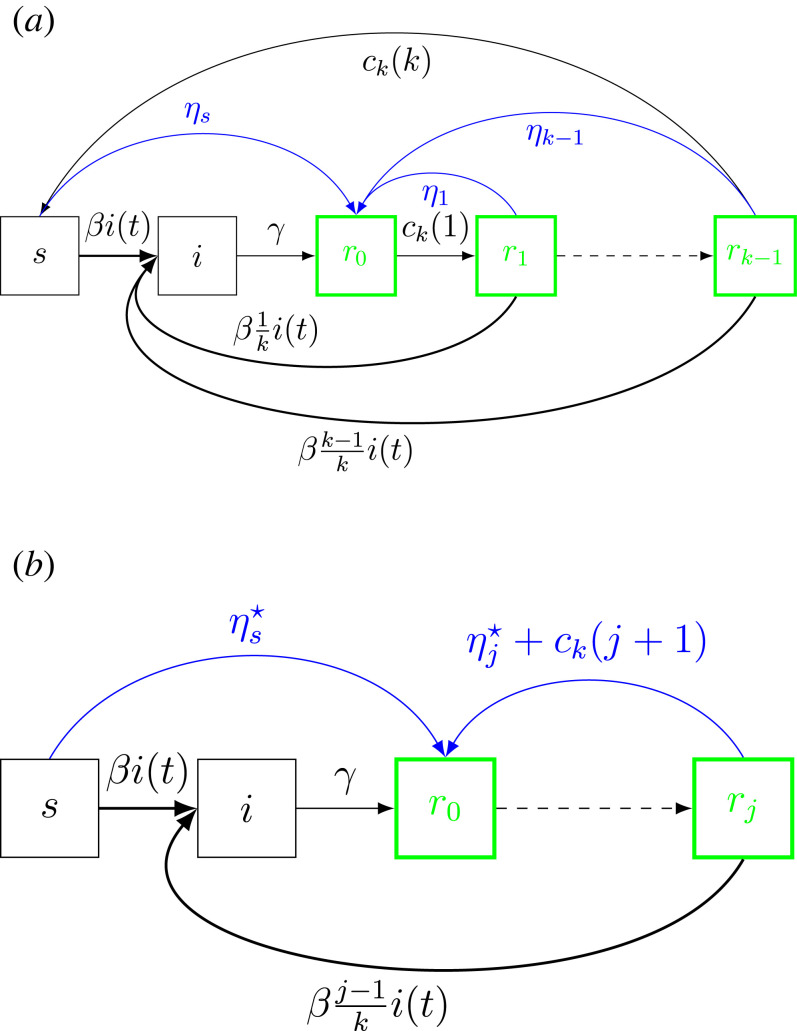
Diagram of SIR^(*k*)^S epidemic model with vaccination. (*a*) General vaccination scheme where partially susceptible individuals *r*_*j*_ are vaccinated at rate *η*_*j*_ for any *j* = 1, …, *k* − 1, respectively. (*b*) Rational vaccination scheme where fully susceptible individuals are vaccinated at rate $\eta_{s}^{⋆}$ and, if needed, only one class *r*_*j*_ of partially susceptible individuals is vaccinated at rate $\eta_{\;j}^{⋆}+c_{k}(j+1)$ for some *j* ∈ {1, …, *k* − 1}.

In the case where immunity wanes continuously, vaccines can in principle be distributed in many different ways (figure 3*a*). However, since individuals only differ in terms of susceptibility in our model, it should be clear that the class of rational vaccination strategies consist of vaccinating individuals as soon as their immunity drops below some fixed level ι (or equivalently when the susceptibility reaches the level 1−ι). The level ι will determine how much vaccine will be required: the larger ι the bigger vaccine supply *θ* is needed. For finite *k* this amounts to vaccinate fully susceptible individuals at rate $\eta_{s}^{⋆}$, to not vaccinate in states *r*_0_ up to *r*_*j*−1_ for some *j* ∈ {1, …, *k* − 1}, to vaccinate *r*_*j*_ at some rate $\eta_{\;j}^{⋆}$, and to immediately vaccinate individuals who go from state *r*_*j*_ to *r*_*j*+1_ (so the fractions in those states will equal 0). Since individuals in state *r*_*j*_ who lose more immunity are immediately vaccinated, the effective vaccination rate in this class equals $\eta_{\;j}^{⋆}+c_{k}(j+1)$. Figure 3*b* represents the corresponding SIR^(*k*)^S model with such vaccination scheme. An important question is hence to determine how much vaccine supply *θ*_*c*_ (critical vaccine supply) is needed to reach and maintain herd immunity.

### 2.2. Parameter choices

In what follows, we will compare the three models in terms of steady-state prevalence (endemic level) if no prevention measures are put in place, and how much vaccine is required to reach and maintain herd immunity. In table 1 we show the mid-value and range for the model parameters that are used in §2.3 when comparing the classic SIRS model with our new models having linear and exponential waning of immunity respectively. Those values are commonly used to characterize diseases like COVID-19, influenza, common cold, etc. [20–23]. Average life expectancy is set at 80 years. Specifically, one can also use empirical data as in [4,6] to infer not only the waning rate *ω* but also the immunity waning function. Please note that the results are not applicable to certain childhood diseases where immunity typically is close to lifelong (measles, chickenpox, …).

**Table 1.** Parameters description, their baseline values and ranges of variation studied.

parameter	description	baseline value	range
*R*_0_	basic reproduction number	5	1–7
*γ*^−1^	mean infectious period (in days)	7	3–14
*ω*^−1^	average immune period (in months)	12	6–24

### 2.3. Main results

We now compare the three epidemic models, the classic SIRS, the model with linearly waning of immunity, and the model with exponentially decaying immunity, all three models being calibrated by having the same cumulative immunity. Some analytical results are obtained for *k* = 2 (see the electronic supplementary material) and conjectured to any *k* > 2.

#### 2.3.1. The basic reproduction number *R*_0_

The basic reproduction number, defined as the number of secondary cases produced by one infectious individual in a fully susceptible population, equals *R*_0_ = *β*/(*γ* + *μ*) for the classic SIRS model (2.1) as well as for our extended models (§4.1). In fact, the models differ only in terms of how immunity wanes and in the initial phase of the epidemic, when nearly everyone is susceptible, immunity waning has no impact. From now on, we assume that *R*_0_ > 1—otherwise none of the three models will experience any outbreak and hence vaccination is not necessary.

#### 2.3.2. Long-term prevalence in the absence of vaccination

**Figure 4.**
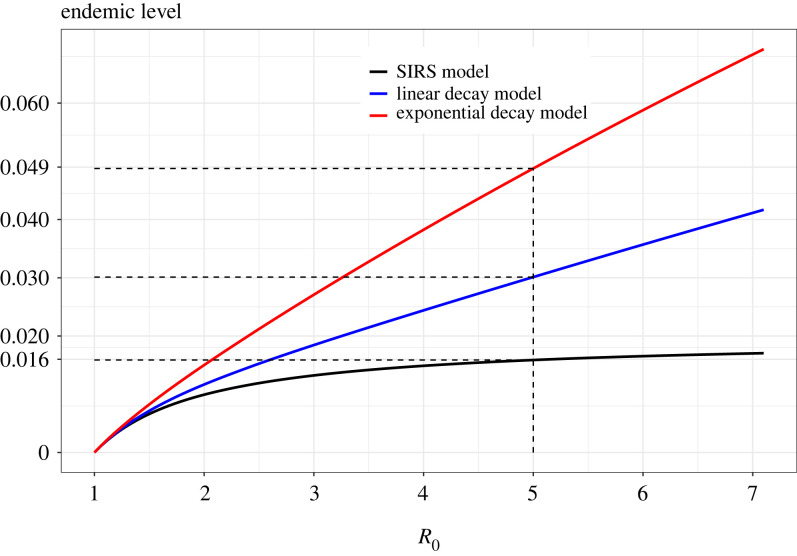
Endemic levels from the standard model and the SIR^(*k*)^S model with linear and exponential waning functions.

A comparison of long-term prevalence is obtained by setting the defining differential equations for each of the three models (given in §4) equal to 0 and solving the equation system. When *R*_0_ > 1, there is one stable solution with a positive infectious fraction $\bar{i}$, the endemic level or the stable prevalence. In figure 4, these endemic levels are given for the three models as a function of *R*_0_ (keeping the mean infectious period and average cumulative immunity fixed). It can be seen that the model with linear waning of immunity results in larger endemic levels compared with the SIRS epidemic. The model with exponential waning of immunity makes the long-term prevalence even larger. When *R*_0_ ≈ 5 as for COVID-19 Delta strain (e.g. [23]) and with a mean infectious period of 7 days and an average duration of immunity of 1 year, the stable prevalence will consist of 1.6% being infectious according to the SIRS model. The linear waning model has about twice the endemic level (3% of the population) and the model with exponential waning has stable prevalence 4.9% (figure 4).

This observation, that gradual waning of immunity (instead of the more extreme situation with individuals being either fully immune or fully susceptible) results in a higher endemic level, is in line with other results on heterogeneity of susceptibility in epidemic modelling. For example, Ball [24] shows that when the (static) susceptibility varies in the population, then fewer people will get infected when compared with all individuals having equal susceptibility (set to the mean susceptibility in the heterogeneous situation). The heterogeneous situation is in this sense ‘better’, and slightly extended, the more heterogeneous the better. In the present work, the SIRS model is the most heterogeneous of all immunity waning models: each individual is either fully immune or fully susceptible. When considering gradual waning of immunity, the variation/heterogeneity in immunity is clearly smaller since immunity now is continuously distributed between full and no immunity. The extension to gradual waning hence makes the model *less* heterogeneous which hence should make the situation worse, meaning a higher prevalence.

#### 2.3.3. Critical vaccine to reach and maintain herd immunity

**Figure 5.**
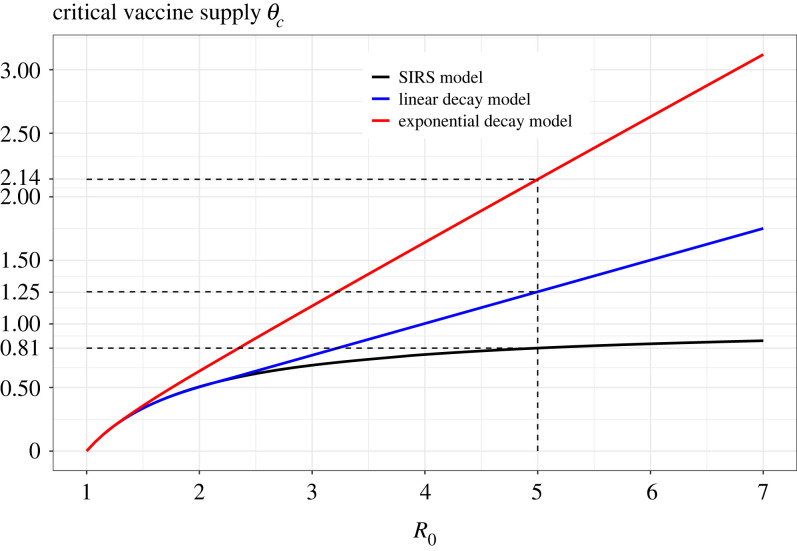
Critical amount of vaccines needed to reach herd immunity.

In figure 5, we show the necessary amount of vaccine supply (for the three models) continuously needed to reach and maintain herd immunity (see §4 for the derivation). It is seen that the standard SIRS model requires a lower vaccine supply as compared with the two models with gradual waning, and that the model with exponential immunity waning requires the largest vaccine supply. Moreover, the difference between the three models grows with *R*_0_. Take as illustration *R*_0_ ≈ 5, a mean infectious period of 7 days, and a 1-year cumulative immunity (inspired by COVID-19 pandemic Delta strain), then the classic SIRS model requires vaccinating at rate 0.81 to reach herd immunity (so 8.1 million vaccinations per year in a population of 10 million), the model with linear waning requires 1.25, and the exponential waning model requires 2.14, some 55% and 164% more vaccinations, respectively. Also here our conclusion is that switching from the most variable/heterogeneous immunity model (SIRS), only having full or no immunity, to the new model with gradual waning, reduces variability/heterogeneity and thus makes the situation worse by requiring more frequent vaccination of individuals, thus requiring more vaccines. This finding is hence in line with previously established results stating that heterogeneity of susceptibility reduces the critical vaccination coverage [25,26].

#### 2.3.4. Comparison of the models for endemic diseases

For new emerging diseases, *R*_0_ is often estimated from the initial growth rate of the epidemic (together with knowledge about the generation time of the disease) [27]. Then the natural calibration of models was to assume the same cumulative immunity *ω*^−1^, and the same transmission rate *β*, recovery rate *γ* as done above.

**Figure 6.**
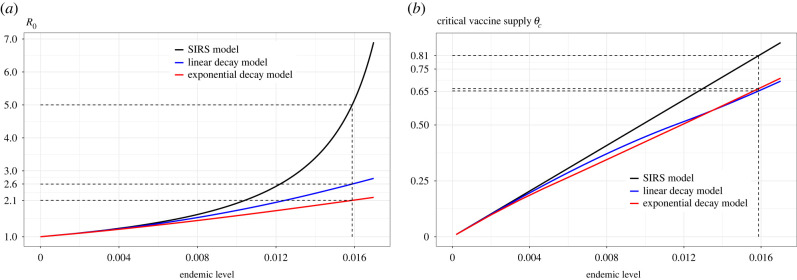
(*a*) *R*_0_ estimates from prevalence (endemic) data and (*b*) the corresponding amount of vaccines needed to reach herd immunity.

For diseases that are currently endemic, a more natural calibration is instead to assume the different models have the same cumulative immunity *ω*^−1^ and the same recovery rate *γ*, and that the endemic level equals the empirical level (so fixing the endemic level rather than *R*_0_). An argument for this calibration is that, while immunity duration and infectious period may be easy to estimate, the same is usually not true for the rate of infectious contacts *β*, which in turn determines *R*_0_. Figure 6*a* shows the estimated *R*_0_ for the different models based on such a calibration, for different values of the endemic level (stable prevalence). The estimate based on the SIRS epidemic was derived in [28].

As seen in the figure, the SIRS model results estimate *R*_0_ being larger than the models with gradual waning, and in particular compared with the model with exponential waning. When the endemic level (prevalence) is 1.6% of the population, the exponential waning model and the linear waning model estimate *R*_0_ to 2.1 and 2.6, respectively, which are 58% and 48% less than the value 5 estimated by the classic SIRS model. Yet, when the prevalence is low, all models result in approximately the same *R*_0_.

If we instead use the endemic level (together with fixing cumulative immunity and the infectious period) to estimate the required amount of vaccine supply for the different models, then the classic SIRS model requires higher amounts of vaccines. This is because the disease is spreading faster in the SIRS setting than in the models with gradual waning. This is illustrated in figure 6*b* which shows that slightly less vaccine supply is required to reach herd immunity under the SIR^(∞)^S models in comparison with the standard SIRS model, and this applies for both linear and exponential waning functions of immunity (19% less vaccines if the disease persists in 1.6% of the population).

## 3. Discussion

The classic SIRS model assumes that immunity at the individual level is binary, i.e. each individual is either fully immune or fully susceptible. This paper relaxes this assumption by presenting and analysing a novel model allowing for gradual waning of immunity, either linear waning or exponential waning. It is shown that, when calibrating the models by assuming the same *R*_0_, mean infectious period and cumulative immunity, the new more realistic models result in higher endemic levels if prevention is not put in place, and that a substantially larger vaccine supply is required to reach and sustain herd immunity. The most realistic model having exponential waning of immunity is shown to exhibit the biggest difference compared with the classic SIRS model.

The studied model can in principle be defined also for other forms of deterministic immunity waning functions. However, it is not known how these waning functions can be mapped from antibody titres that decay over time. While immunity could wane at once, this paper shows how big the difference is assuming continuous waning.

Most often newly developed models introduce more heterogeneity, and when compared with earlier more homogeneous models hence often giving a less severe situation (fewer infected, lower vaccination coverage needed,…), e.g. [24–26]. Here, our extension goes in the opposite direction: the effect of considering gradual waning of immunity when compared with having a 0–1-model for immunity actually makes the model more *homogeneous*, which explains why the situation becomes worse. In some sense, other heterogeneities not taken into account in a model may hence make the situation better again. The magnitude of this latter effect is of course hard to determine.

Our model extends the SIRS epidemic to allow for gradual waning of immunity. Many other model assumptions are admittedly unrealistic and oversimplify various immunity aspects. It would of course be interesting to study this extension to gradual waning of immunity when e.g. not obtaining full immunity from start or combining leaky vaccine with continuous waning, having different forms of immunity waning for vaccine when compared with disease exposure, considering asymptomatic and symptomatic individuals. The effect that seasonality in combination with gradual waning has on suitable vaccination policies is another important open question. Still it our belief that the qualitative feature, that gradual waning requires bigger vaccine supply, remains.

Another assumption was that the immunity status of individuals were known when determining whom to vaccinate. In the case where immunity wanes deterministically, as in the two new models, this might be a reasonable approximation since the time of last vaccination or infection might be known, but when immunity wanes in one leap after an exponential time, this may not be possible. Analysing models where the exact immunity status is unknown, incorporating variation in individuals’ immunity waning profiles, perhaps by introducing randomness in waning decay, is an interesting problem to analyse. In real life, exposure to a disease may boost an individual’s immunity without the individual getting seriously infected. Including such boosting into a model would be another interesting extension of the current work.

## 4. Methodology and proofs

We summarize the methods used to establish the results listed in §2.3. We start by formulating the SIR^(*k*)^S model with gradual waning of immunity including linear and exponential decaying functions. A rigorous mathematical analysis of the model is given in the case *k* = 2 (see the electronic supplementary material), thus, allowing to make conjecture for any *k* > 2.

### 4.1. General SIR^(*k*)^S epidemic model

The general SIR^(*k*)^S model (figure 2*b*) introduced in this paper aims to approximate the linear and exponential immunity waning modes (figure 1) using step functions such that all immunity is lost after *k* jumps, starting by complete immunity to zero immunity, losing a portion 1/*k* each step. Here, we outline how to construct the SIR^(*k*)^S model following any function of waning of immunity.

Suppose that for a given decaying function and an arbitrary integer *k* ≥ 1, immunity level (*k* − *j*)/*k* lasts for an exponentially distributed time with rate *c*_*k*_(*j* + 1) > 0 before decaying to ((*k* − *j*)/*k*) − (1/*k*) with *j* going from 0 to *k* − 1, such that the rates ${\left\{c_{k}(j)\right\}}_{\;j=1}^{k}$ verify the constant cumulative immunity condition

4.1 $$\sum_{\;j=0}^{k-1}\frac{1}{c_{k}(j+1)}\left(1-\frac{\;j}{k}\right)=\frac{1}{\omega },$$

and approximate the underlying waning of immunity. Denote by ${\left\{r_{\;j}(t)\right\}}_{\;j=0}^{k-1}$ the fractions of individuals (at time *t*) with the immunity level (*k* − *j*)/*k* (or equivalently, with susceptibility level *j*/*k*). Infectious individuals that recover enter the highest (= full) immunity class, *r*_0_(*t*), and then their immunity declines through *k* steps. The class of individuals *r*_*k*−1_(*t*) has the lowest immunity level, 1/*k*, to be lost altogether to become fully susceptible again. Thus, the resulting SIR^(*k*)^S model with gradual waning of immunity can be formulated as in the following equations:

4.2 $$\begin{array}{rl} s^{\prime }(t) & =\mu -\beta s(t)i(t)+c_{k}(k)r_{k-1}(t)-\mu s(t),\\ i^{\prime }(t) & =\beta s(t)i(t)+\beta \sum_{\;j=1}^{k-1}\frac{\;j}{k}r_{\;j}(t)i(t)-(\gamma +\mu )i(t),\\ r_{0}^{\prime }(t) & =\gamma i(t)-(c_{k}(1)+\mu )r_{0}(t)\\ \mathrm{and}\;\;r_{\;j^{\prime }}(t) & =c_{k}(j)r_{\;j-1}(t)-\beta \frac{\;j}{k}r_{\;j}(t)i(t)-(c_{k}(j+1)+\mu )r_{\;j}(t), \end{array}\}$$

for *j* = 1, …, *k* − 1, where we omit the dependence on *k* in (*s*(*t*), *i*(*t*), *r*_0_(*t*), …, *r*_*k*−1_(*t*)) for simplicity of notation. We will also use the notation $\left(\hat{s},0,{\hat{r}}_{0},\ldots ,{\hat{r}}_{k-1}\right)$ for the disease-free equilibrium and $\left(\bar{s},\bar{i},{\bar{r}}_{0},\ldots ,{\bar{r}}_{k-1}\right)$ for the endemic equilibrium.

The disease-free equilibrium (DFE) of the model (4.2) is *E*^*k*,0^ = (1, 0, …, 0). Since the entire population is fully susceptible at the DFE, the initial behaviour of the epidemic is governed by the sign of *β* − (*γ* + *μ*). This leads to the definition of the basic reproduction number

$$R_{0}=\frac{\beta }{\gamma +\mu }.$$

The sequence ${\left\{c_{k}(j)\right\}}_{\;j=1}^{k}$ is chosen to fit the required waning mode of immunity and the fixed cumulative immunity condition (4.1), regardless of *k*.

### 4.2. SIR^(*k*)^S models with linear and exponential waning of immunity

The linear and the exponential functions modelling the waning of immunity (figure 1) given by

4.3 $$\begin{array}{r} g(u)=\left(1-\frac{\omega }{2}a\right)1_{\left\{a<(2/\omega )\right\}}\;\mathrm{and}\;h(u)=\exp(-\omega u),\;u\geq 0, \end{array}$$

respectively, with the indicator function **1**_*A*_ equal to 1 if the condition *A* holds and 0 otherwise, verify the same cumulative immunity condition

$$\int_{R^{+}}g(u)\;du=\int_{R^{+}}h(u)\;du=\frac{1}{\omega },$$

which is equal to the average cumulative immunity from the standard SIRS model with immunity waning rate *ω*. To parametrize the model fitting *g*, one can assume constant transition rates that verify the condition (4.1). This leads to

4.4 $$c_{k}(j)=\frac{k+1}{2}\omega ,\;j=1,\ldots ,k.$$

We refer to the model (4.2) with the rates given by (4.4) as the SIR^(*k*)^S model with linear waning of immunity. A way to fit the exponential waning function *h* is to choose a decreasing transition rates sequence satisfying both the condition (4.1) and

4.5 $$\sum_{l=1}^{\;j}\frac{1}{c_{k}(l)}=h^{-1}\left(1-\frac{\;j}{k}\frac{k-1}{k}\right),\;j=1,\ldots ,k-1.$$

Hence, one can define

**Figure 7.**
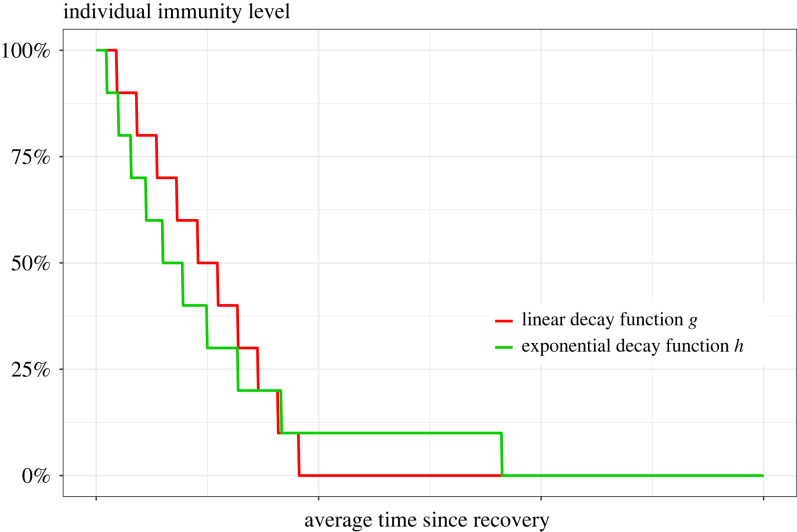
Step functions approximating the immunity waning functions *g* and *h* in the case *k* = 10 steps.

4.6 $$\begin{array}{rl} c_{k}(j) & ={\left(-\frac{1}{\omega }\log(1-j(k-1)/k^{2})-\sum_{l=1}^{\;j-1}\frac{1}{c_{k}(l)}\right)}^{-1},\\ j & =1,\ldots ,k-1, \end{array}$$

and obtain *c*_*k*_(*k*) by solving the equation (4.1). We refer to the corresponding model as the SIR^(*k*)^S model with exponential waning of immunity. Figure 7 plots the corresponding step functions for *k* = 10 and where the duration of each immunity level is set to its expected value.

Different rates ${\left\{c_{k}(j)\right\}}_{\;j=1}^{k}$ other than (4.4) and (4.6) can be considered to approximate the linear and the exponential waning functions, respectively. Still, they have no effect on the model dynamics as *k* → ∞.

### 4.3. SIR^(*k*)^S model with vaccination

We now introduce vaccination into the model and make the simplification that immunity from vaccination is identical to immunity from disease exposure (complete immunity with the same waning mode). The resulting SIR^(*k*)^S model with a general vaccination scheme can be written as

4.7 $$\begin{array}{rl} s^{\eta^{\prime }}(t) & =\mu -\beta s^{\eta }(t)i^{\eta }(t)+c_{k}(k)r_{k-1}^{\eta }(t)-\mu s^{\eta }(t)-\eta_{s}s^{\eta }(t),\\ i^{\eta^{\prime }}(t) & =\beta s^{\eta }(t)i^{\eta }(t)+\beta \sum_{\;j=1}^{k-1}\frac{\;j}{k}r_{\;j}^{\eta }(t)i^{\eta }(t)-(\gamma +\mu )i^{\eta }(t),\\ r_{0}^{\eta^{\prime }}(t) & =\eta_{s}s^{\eta }(t)+\gamma i^{\eta }(t)-(c_{k}(1)+\mu )r_{0}^{\eta }(t)+\sum_{\;j=1}^{k-1}\eta_{\;j}r_{\;j}^{\eta }(t)\\ \mathrm{and}\;r_{\;j}^{\eta^{\prime }}(t) & =c_{k}(j)r_{\;j-1}^{\eta }(t)-\beta \frac{\;j}{k}r_{\;j}^{\eta }(t)i^{\eta }(t)\\ & \;-(c_{k}(j+1)+\mu )r_{\;j}^{\eta }(t)-\eta_{\;j}r_{\;j}^{\eta }(t), \end{array}\}$$

for *j* = 1, …, *k* − 1, where *η*_*s*_ and *η*_*j*_ ≥ 0, *j* = 1, …, *k* − 1 are the rates of vaccination of *s*^***η***^(*t*) and $r_{\;j}^{\eta }(t),\;j=1,\ldots ,k-1$, respectively. The disease-free equilibrium $E_{v}^{k,0}=({\hat{s}}^{\eta },0,{\hat{r}}_{0}^{\eta },\ldots ,{\hat{r}}_{k-1}^{\eta })$ is given by

4.8 $${\hat{s}}^{\eta }=\frac{\mu }{\mu +\eta_{s}-\eta_{s}c_{k}(k)A_{k}B_{k-1}},$$

4.9 $$\;\;\;\;{\hat{r}}_{\;j}^{\eta }=\eta_{s}{\hat{s}}^{\eta }A_{k}B_{\;j},\;j=1,\ldots ,k-1$$

4.10 $$\mathrm{and}\;\;\;{\hat{r}}_{0}^{\eta }=1-{\hat{s}}^{\eta }-\sum_{\;j=1}^{k-1}{\hat{r}}_{\;j}^{\eta },$$

where $B_{\;j}=\prod_{l=1}^{\;j}(c_{k}(l)/\left(\mu +c_{k}(l+1)+\eta_{l}\right)),j=1,\ldots ,k-1$, and $A_{k}={\left(\mu +c_{k}(1)-\sum_{\;j=1}^{k-1}\eta_{\;j}B_{\;j}\right)}^{-1}.$ The effective reproduction number is given by

4.11 $$R^{\eta }=R_{0}\left({\hat{s}}^{\eta }+\sum_{\;j=1}^{k-1}\frac{\;j}{k}{\hat{r}}_{\;j}^{\eta }\right),$$

where we recall that $\frac{\;j}{k}$ is the relative susceptibility in the *j*th immunity state.

### 4.4. Critical vaccine supply

The vaccine usage (per unit of time) for the general vaccination scheme of the previous subsection, once it has reached steady state, is given by

4.12 $$\theta^{\left(k\right)}=\eta_{s}{\hat{s}}^{\eta }+\sum_{\;j=1}^{k-1}\eta_{\;j}{\hat{r}}_{\;j}^{\eta }.$$

For fixed *k*, the best vaccination strategy, given some amount of vaccine supply delivered continuously, is clearly to vaccinate the most susceptible (= least immune) individuals. More precisely, the best strategy is to immediately vaccinate individuals having higher susceptibility than some class *j*, to vaccinate individuals in susceptibility class *j* at rate $\eta_{\;j}^{⋆}$, and to not vaccinate individuals in susceptibility classes lower than *j* (i.e. *r*_0_, …, *r*_*j*−1_), where *j* and $\eta_{\;j}^{⋆}$ will depend on the amount of available vaccine. With this vaccination strategy, individuals moving to state *j* + 1 (with rate *c*_*k*_(*j* + 1)) will be vaccinated immediately and no individuals will ever reach higher susceptibility classes, so the actual vaccination rate among individuals in class *r*_*j*_ is $\eta_{\;j}^{⋆}+c_{k}(j+1)$. Newborns should also be vaccinated, at their incoming rate *μ*, when we vaccinate in the class *r*_*j*_. For this strategy to be successful in the long run the amount of vaccine supply should be such that *j* and $\eta_{\;j}^{⋆}$ are the minimal immunity level and the minimal vaccination rate respectively, satisfying *R*^***η***^ ≤ 1. Hence, the critical vaccine supply can be written as

4.13 $$\begin{array}{c} \theta_{c}^{\left(k\right)}=\underset{\;j}{\min}\;\theta_{\;j}^{\left(k\right)}\\ \mathrm{so}\;\mathrm{that}\;R^{\eta }\leq 1, \end{array}$$

where

4.14 $$\begin{array}{rl} & \theta_{\;j}^{\left(k\right)}=\\ & \left\{ \begin{array}{cc} \eta_{s}^{⋆}{\hat{s}}^{\eta }\; & \text{if we only vaccinate fully susceptibles},\;\\ \mu +(\eta_{\;j}^{⋆}+c_{k}(j+1)){\hat{r}}_{\;j}^{\eta }\; & \text{if we vaccinate in the class }r_{\;j}.\; \end{array}\right. \end{array}$$

A detailed derivation of equation (4.13) when *k* = 2 is given in the electronic supplementary material.

An alternative way to derive the critical vaccine supply is to assume that the disease is in the endemic steady state and then vaccinate in each immunity class and to check that the disease-free equilibrium $E_{v}^{k,0}$ is the only stable steady state.

### 4.5. Standard SIRS epidemic model

It has been shown that the standard SIRS model (2.1) admits a unique endemic equilibrium when *R*_0_ > 1 and only the disease-free equilibrium exists when *R*_0_ ≤ 1 [1]. When susceptibles are vaccinated at a constant rate *η*, the resulting SIRS model has a unique endemic equilibrium when *R*^***η***^ > 1, and only the disease-free equilibrium exists when *R*^***η***^ ≤ 1, where $R^{\eta }=R_{0}\hat{s}$ is the average number of new infections generated by an infective individual in a population with susceptible fraction of $\hat{s}$ [29]. Since $\hat{s}=\left(\mu +\omega \right)/\left(\mu +\omega +\eta \right)$, the minimum vaccination rate to drive the epidemic dynamic to the disease-free state (i.e. at which $R_{0}\hat{s}=1$) is given by

$$\eta_{c}=(\omega +\mu )(R_{0}-1).$$

Hence, the critical vaccine supply required to achieve and maintain the disease-free equilibrium is defined as the product of the rate *η*_*c*_ and the fraction $\hat{s}=1/R_{0}$, which is equal to

4.15 $$\theta_{c}^{\left(1\right)}=(\omega +\mu )\left(1-\frac{1}{R_{0}}\right).$$

### 4.6. Endemic level

The following proposition shows that the SIR^(*k*)^S model (4.2) admits a unique endemic equilibrium if and only if *R*_0_ > 1. This goes in line with the result established in [9] for the limiting model stating that an endemic equilibrium exists if and only if *R*_0_ > 1.

**Proposition 4.1.**

*The* SIR^(*k*)^S *model without vaccination* (*4.2*) *has a unique endemic equilibrium* $E^{k,*}=(\bar{s},\bar{i},{\bar{r}}_{0},\ldots ,{\bar{r}}_{k-1})$ *if and only if* *R*_0_ > 1.

The proof of proposition 4.1 is given in the electronic supplementary material. In the model with vaccination, we only proved that *R*^***η***^ > 1 is a necessary and sufficient condition for the existence of a unique endemic equilibrium to the system (4.7) in the case *k* = 2. We conjecture this to any *k* > 2.

**Proposition 4.2.**

*The* SIR^(2)^S *model* (*4.7*) *with vaccination has a unique endemic equilibrium* $E_{v}^{2,*}=({\bar{s}}^{\eta },{\bar{i}}^{\eta },{\bar{r}}_{0}^{\eta },{\bar{r}}_{1}^{\eta })$ *if and only if* *R*^***η***^ > 1.

**Conjecture 4.3.**

For any *k* > 2, the SIR^(*k*)^S model with vaccination (4.7) has a unique endemic equilibrium $E_{v}^{k,*}=({\bar{s}}^{\eta },{\bar{i}}^{\eta },{\bar{r}}_{0}^{\eta },\ldots ,{\bar{r}}_{k-1}^{\eta })$ if and only if *R*^***η***^ > 1.

### 4.7. Critical immunity level

**Figure 8.**
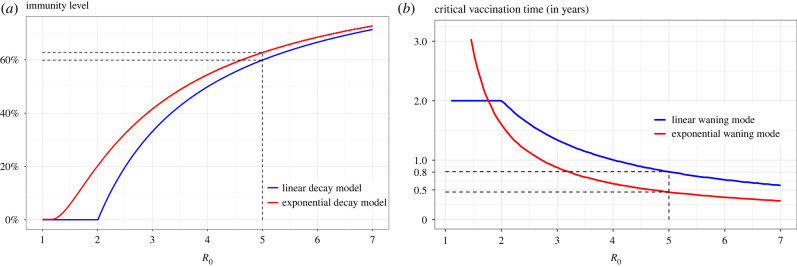
(*a*) Immunity level ι at which individuals have to be vaccinated (at the very latest), as a function of *R*_0_ and (*b*) the corresponding vaccination time, using baseline parameter values in table 1.

Clearly, the critical immunity level ι, at which individuals have to be vaccinated in order to reach herd immunity from the limiting SIR^(*k*)^S models with linear and exponential decays of immunity, is defined by ι=1−j/k, where *j* is the integer defining the optimal vaccination strategy given in equation (4.13). Figure 8*a* plots ι as a function of *R*_0_. We recall that the classic SIRS model assumes all individuals are either completely immune or completely susceptible, something which is not true in the models for gradual immunity waning. For parameter choices resembling the COVID-19 Delta strain (*R*_0_ ≈ 5, *ω*^−1^ = 12 months and *γ*^−1^ = 7 days), herd immunity will only be achieved if individuals are vaccinated before their immunity drops below ι≈60% , according to the SIR^(*k*)^S models with linear and exponential decays of immunity. This also means that individuals should get booster vaccines approximately every 10 months and 6 months since their last vaccination/infection when immunity wanes linearly and exponentially, respectively (figure 8*b*).

### 4.8. Connection with ODE-PDE model

As *k* → ∞, the number of states in the SIR^(*k*)^S model increases and there is a continuity of immunity states. This limiting model can be described by an ODE-PDE system. Since we are interested in deterministic linear and exponential waning of immunity, knowing an individual’s immunity level is equivalent to knowing the amount of time since his last recovery: time since recovery. The corresponding models can be formulated as follows.

#### 4.8.1. Linear waning model

Assume a continuous linear waning of immunity (§4.2) and let *r*(*t*, *a*) to be the fraction (density) of recovered individuals at time *t* with age *a* since recovery. For an infinitesimal time step d*t*, the individuals in *r*(*t*, *a*) are those among *r*(*t* − d*t*, *a* − d*t*) who will neither die nor get infected during the interval time [*t*, *t* + d*t*], that is, we have for any *a* > 0,

4.16 $$\begin{array}{rl} r(t,a) & =r(t-dt,a-dt)\left(1-\underset{\text{death rate}}{\underbrace{\mu }}\;dt\right)\\ & \;\times \left(1-\underset{\text{infection rate}}{\underbrace{\beta \left(\left(\frac{\omega }{2}a-1\right)1_{\left\{a<(2/\omega )\right\}}+1\right)\;i(t)}}\;dt\right), \end{array}$$

and

4.17 r(t,0)=γi(t).

Rearranging the equation (4.16) and sending d*t* to 0, it yields that

4.18 $$\begin{array}{r} \frac{\partial r(t,a)}{\partial t}+\frac{\partial r(t,a)}{\partial a}=-\beta \left(\left(\frac{\omega }{2}a-1\right)1_{\left\{a<\frac{2}{\omega }\right\}}+1\right)r(t,a)i(t)-\mu r(t,a). \end{array}$$

Then, the PDE-ODE model has the following form

4.19 $$\begin{array}{rl} & s^{\prime }(t)=\mu -\beta s(t)i(t)-\mu s(t),\\ & i^{\prime }(t)=\beta \left(s(t)+\int_{2/\omega }^{\infty }\;r(t,\tau )\;d\tau \;\right)i(t)\\ & \;\;+\beta \int_{0}^{2/\omega }\frac{\omega }{2}\tau r(t,\tau )\;d\tau \;i(t)-(\gamma +\mu )i(t),\\ \mathrm{and}\; & \frac{\partial r(t,a)}{\partial t}+\frac{\partial r(t,a)}{\partial a}=-\beta \left(\left(\frac{\omega }{2}a-1\right)1_{\left\{a<(2/\omega )\right\}}+1\right)\\ & \;r(t,a)i(t)-\mu r(t,a),\;a>0, \end{array}\}$$

with the boundary condition *r*(*t*, 0) = *γi*(*t*).

#### 4.8.2. Exponential decay model

Similarly to the previous paragraph, the corresponding PDE-ODE model when immunity wanes exponentially can be written as

4.20 $$\begin{array}{rl} & s^{\prime }(t)=\mu -\beta s(t)i(t)-\mu s(t),\\ & i^{\prime }(t)=\beta s(t)i(t)+\beta \int_{0}^{\infty }(1-e^{-\omega \tau })\;r(t,\tau )\;d\tau \;i(t)-(\gamma +\mu )i(t)\\ \mathrm{and}\; & \frac{\partial r(t,a)}{\partial t}+\frac{\partial r(t,a)}{\partial a}=-\beta (1-e^{-\omega a})\;r(t,a)i(t)-\mu r(t,a),\;a>0, \end{array}\}$$

with the boundary condition *r*(*t*, 0) = *γi*(*t*).

Similar models to equations (4.19) and (4.20) but with general waning rates were previously introduced and analysed in the literature, e.g. [12]. It has been shown in [30] that each of the models (4.19) and (4.20) has only a disease-free equilibrium (which is globally asymptotically stable) when the basic reproduction number *R*_0_ ≤ 1, and a unique locally stable endemic equilibrium when *R*_0_ > 1. We refer to the seminal works of Kermack & McKendrick [19,31] and the revisiting paper of Inaba [9] for a general formulation in the case where both virgin and recovered individuals have varying susceptibility and infected ones have variable infectivity. See also [32] where the authors consider the effects of previous infections on the susceptibility of partially susceptible individuals.

## Data Availability

This work did not require ethical approval from a human subject or animal welfare committee.

## Data accessibility

The data are provided in electronic supplementary material [33].
